# Influence of Adaptive Statistical Iterative Reconstructions on CT Radiomic Features in Oncologic Patients

**DOI:** 10.3390/diagnostics11061000

**Published:** 2021-05-31

**Authors:** Damiano Caruso, Marta Zerunian, Francesco Pucciarelli, Benedetta Bracci, Michela Polici, Benedetta D’Arrigo, Tiziano Polidori, Gisella Guido, Luca Barbato, Daniele Polverari, Antonella Benvenga, Elsa Iannicelli, Andrea Laghi

**Affiliations:** Radiology Unit, Department of Medical Surgical Sciences and Translational Medicine, Sapienza University of Rome, Sant’Andrea University Hospital, Via di Grottarossa, 1035-1039, 00189 Rome, Italy; damiano.caruso@uniroma1.it (D.C.); marta.zerunian@uniroma1.it (M.Z.); francesco.pucciarelli@uniroma1.it (F.P.); benedetta.bracci@uniroma1.it (B.B.); michela.polici@uniroma1.it (M.P.); benedetta.darrigo@uniroma1.it (B.D.); tiziano.polidori@uniroma1.it (T.P.); gisella.guido@uniroma1.it (G.G.); lucabarbatotsrm@gmail.com (L.B.); daniele.polverari@gmail.com (D.P.); geltrude.a@gmail.com (A.B.); elsa.iannicelli@uniroma1.it (E.I.)

**Keywords:** iterative reconstruction, filtered back projection, texture analysis, reproducibility

## Abstract

Iterative reconstructions (IR) might alter radiomic features extraction. We aim to evaluate the influence of Adaptive Statistical Iterative Reconstruction-V (ASIR-V) on CT radiomic features. Patients who underwent unenhanced abdominal CT (Revolution Evo, GE Healthcare, USA) were retrospectively enrolled. Raw data of filtered-back projection (FBP) were reconstructed with 10 levels of ASIR-V (10–100%). CT texture analysis (CTTA) of liver, kidney, spleen and paravertebral muscle for all datasets was performed. Six radiomic features (mean intensity, standard deviation (SD), entropy, mean of positive pixel (MPP), skewness, kurtosis) were extracted and compared between FBP and all ASIR-V levels, with and without altering the spatial scale filter (SSF). CTTA of all organs revealed significant differences between FBP and all ASIR-V reconstructions for mean intensity, SD, entropy and MPP (all *p* < 0.0001), while no significant differences were observed for skewness and kurtosis between FBP and all ASIR-V reconstructions (all *p* > 0.05). A per-filter analysis was also performed comparing FBP with all ASIR-V reconstructions for all six SSF separately (SSF0-SSF6). Results showed significant differences between FBP and all ASIR-V reconstruction levels for mean intensity, SD, and MPP (all filters *p* < 0.0315). Skewness and kurtosis showed no differences for all comparisons performed (all *p* > 0.05). The application of incremental ASIR-V levels affects CTTA across various filters. Skewness and kurtosis are not affected by IR and may be reliable quantitative parameters for radiomic analysis.

## 1. Introduction

In the past years the CT field of interest has moved from qualitative to quantitative imaging, especially in oncology scenarios where heterogeneity within solid tumors is usually associated with malignant biology [1].

Among CT quantitative methods explored, CT texture analysis (CTTA) is an area of radiomics that allows an ultrastructural quantitative evaluation by analyzing pixel or voxel grey levels of the image, reflecting the heterogeneity related to the biologic microenvironment [2]. CTTA showed a promising role, especially in oncology, including lesion characterization, response to treatment and prognosis [1,3,4,5,6,7]. 

Despite texture analysis being a promising tool for quantitative assessment of images, due to the novelty of the technique applied in medical imaging, CTTA still needs to be standardized and validated. An important aspect is the CTTA reproducibility related to the influence of CT acquisition parameters (e.g., level of radiation dose, slice thickness, reconstruction algorithms) that can affect results and standardization among different studies [8,9,10]. This aspect is gaining interest, as shown by Erdal et al. [11] who demonstrated that slice thickness influenced reproducibility of radiomic features in lung nodules, and Prezzi et al. [12] who showed the influence of iterative reconstruction (IR) algorithm versus traditional filtered back projection (FBP) on radiomics quantification in twenty-eight datasets of colorectal cancer.

Radiomics studies up to now available in the literature regard mainly analysis on images reconstructed with FBP. 

Nowadays, the IR algorithm has been introduced by all different vendors in CT scanners because it has the advantages of reducing artefacts and noise scanning at lower radiation dose [13,14]. These algorithms represent an extraordinary method to reduce radiation dose and preserving image quality, in particular for oncologic patients and cardiovascular imaging [15,16,17,18]. This technology is evolving rapidly with improved, newer IR methods becoming available. The current version of iterative reconstruction from GE Healthcare is named Adaptive Statistical Iterative Reconstruction-V (ASIR-V) (GE Healthcare, Waukesha, WI, USA), a hybrid technique between the technologies of ASIR and Veo. ASIR-V uses a less complex system model for forward projection, which results in faster reconstruction times, a higher spatial resolution and an additional radiation dose reduction of 35% in the abdomen [19,20]. The ASIR-V technique allows to blend FBP and statistical iterative reconstruction information with increments of 10%, on a scale of 0% to 100%. As an example, ASIR-V 40 means that the algorithm blends 40% ASIR-V with 60% FBP. For a higher ASIR contribution, an image noise reduction is obtained [21,22].

As abovementioned, some studies have demonstrated the influence of IR (i.e., ASIR) on radiomics, but, up to now, no studies have investigated the influence of different incremental levels of ASIR-V on CT radiomic features compared to FBP.

Therefore, the purpose of this study is to evaluate the influence between FBP and different adaptive statistical iterative reconstruction levels (ASIR-V), applied with incremental weightings, on CT radiomic texture features to assess its clinical and technical reproducibility.

## 2. Materials and Methods

### 2.1. Study Population

This retrospective study was conducted according to Declaration of Helsinki guidelines, and written informed consent was obtained from all study participants. From January 2020 to September 2020, unenhanced abdomen CTs of patients that underwent oncological staging were selected.

For the specific purpose of the study, only unenhanced abdomen CTs performed for oncologic staging with the following inclusion criterion were selected: abdominal CT performed with the same multidetector scanner and protocol. Patients with motion or beam hardening artefacts on abdomen CT were excluded.

**Figure 1:**
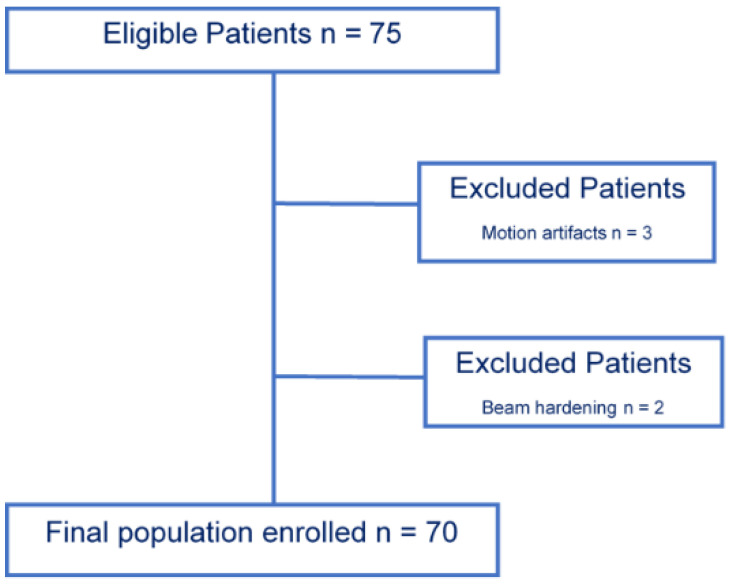
Population enrollment flow-chart. A total of 70 patient were included from an initial population of 75 patients enrolled; three were excluded in relation to motion artefacts, and two were excluded due to beam hardening artifacts.

The study population enrollment flow-chart is summarized in Figure 1.

### 2.2. CT Protocol

All CT exams were performed with a 128-slice CT scanner (GE Revolution EVO CT Scanner, GE Medical Systems, Milwaukee, WI, USA). CT scans were acquired in the cranio-caudal direction with patients placed in a supine position in full end-inspiration with hands above the head. Patients were scanned from the xiphoid process to the pelvic floor. Considering the oncologic population enrolled, multiphase CT examinations were performed (unenhanced, arterial phase, portal phase and delayed phase), but only the unenhanced phase was used for the purpose of the analysis. Internal institution standard acquisition protocol was applied with scan parameters as follows: tube voltage 100 kV, tube current modulation 200–480 mAs, spiral pitch factor 0.98, collimation width 0.625 and gantry rotation time 0.6 s.

### 2.3. Data Reconstruction

**Figure 2:**
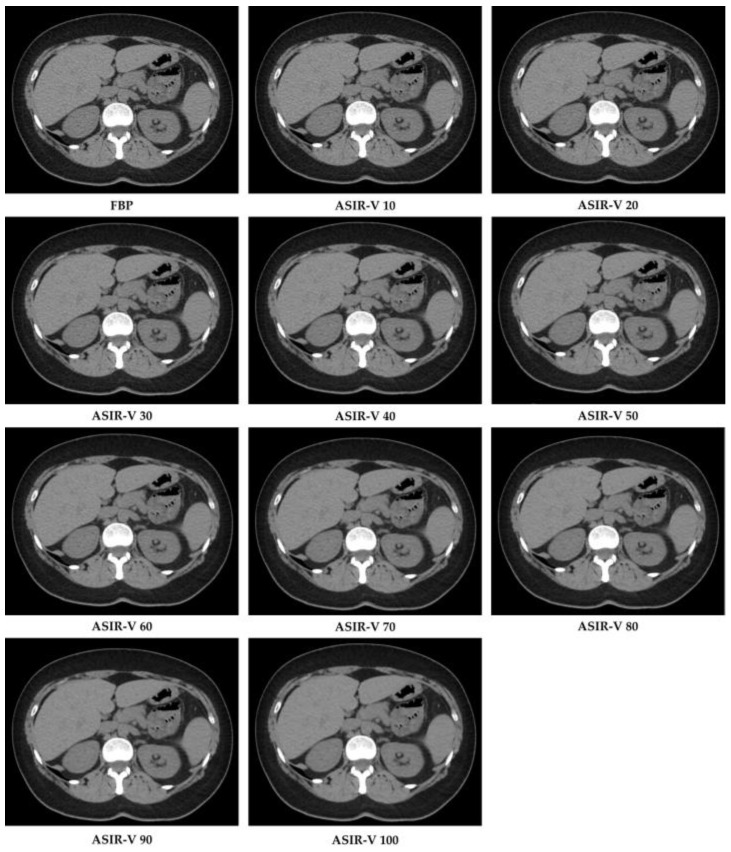
CT image reconstructions at filtered back projection (FPB) and at different Adaptive Statistical Iterative Reconstruction-V (ASIR-V) levels.

Raw data were reconstructed by choosing two algorithms: FBP and ten different ASIR-V levels (from 10% to 100% with incremental factor of 10), with a slice thickness of 1.25 mm and by using an image matrix of 512 × 512 pixels with the standard kernel. In total, this resulted in 11 image datasets per patient (Figure 2).

FBP was the first reconstructive procedure used in CT because of its speed and ease of execution [23]. FBP exploits a mathematical method to reconstruct data using algorithms that solve continuous functions. In this process the intensity values measured by detectors are transformed into mathematical functions (projection data) that are solved and propagated by reconstruction algorithms in a process called “back projection”. Then, projection data are filtered to eliminate the blur effect during the CT reconstruction. FBP has the disadvantage that increasing the sharpness of the image also increases image noise and artefacts [13,24].

After all datasets were completed with both FBP and ASIR-V reconstructions, Digital Imaging and COmmunications in Medicine (DICOM) image data were anonymized and transferred into a picture archiving and communication system (PACS) workstation (Centricity Universal Viewer v.6.0, GE Medical Systems, Milwaukee, WI, USA).

### 2.4. Radiomic Analysis

DICOM images extracted from PACS were transferred to a dedicated texture analysis software (TexRAD, Feedback Medical Ltd., Cambridge, UK). Two radiologists (FP and DC, with 5 and 10 years of experience in abdominal imaging, respectively), in consensus reading, chose a single slice of the unenhanced CT where liver, kidneys, spleen and paravertebral muscles were clearly represented and drew a fixed circular region of interest (ROI) (area 1 cm^2^) on each structure assessed (liver, right kidney, spleen and left paravertebral muscle) with fixed abdomen window (width: 400 HU; level: 40 HU). Every ROI included only parenchymal structures, excluding from the analysis other structures (e.g., vessels, focal lesions). ROIs were cloned for all different reconstruction datasets (FBP and ASIR-V from 10% to 100%), and then CTTAs were extracted from all datasets.

**Figure 3:**
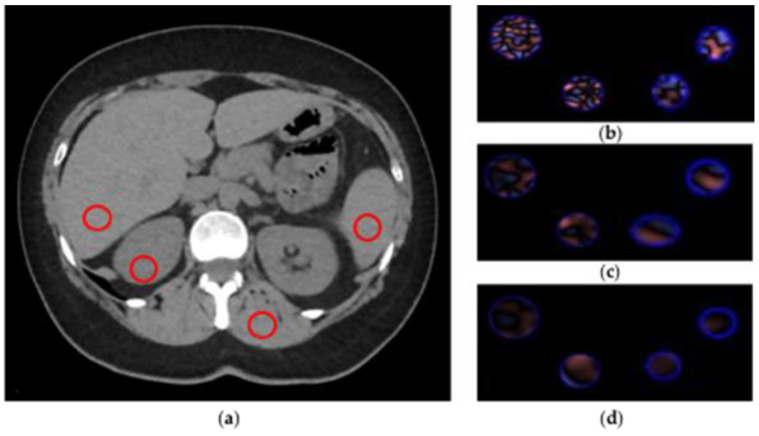
CT texture analysis (CTTA) process. (**a**) Regions of interest (ROIs) placement. (**b**–**d**) Filtration-histogram statistic-based method corresponding to fine, medium and coarse texture scale of ROIs (area 1 cm^2^), respectively.

CTTA extracted from abdominal CT is a first-order radiomic feature based on a statistical method that quantifies the heterogeneity of a ROI, analyzing the intensity of pixel frequency and then extracting the following histogram parameters: mean intensity, standard deviation, entropy, kurtosis, skewness, and mean value of positive pixels (MPP). For CTTA, spatial filters, denoted by spatial scaling factors (SSFs) of 0 to 6, were applied. SSF 0 indicates no filtration; SSFs 2, 4 and 6 indicate 2, 4 and 6 mm radii, which represent fine, medium and coarse filters, respectively. These filters analyze the ROI at a different scale with object radii of different sizes. All the parameters extracted were assessed with and without altering the SSF with a Laplacian of Gaussian spatial band-pass filter to highlight features at different anatomic spatial scales at fine, medium and coarse texture. Figure 3 shows the filtration-histogram-based CTTA of FBP reconstruction and ASIR-V 100.

### 2.5. Statistical Analysis

Categorical variables are given as numbers and percentages and continuous variables as mean and standard deviation. The Kolmogorov–Smirnov test was performed to establish normality.

Paired t-test and Wilcoxon matched paired test were performed to assess the ability of CTTA to differentiate between FBP and all ASIR-V levels. In particular, the paired t-test was used for parametric samples and Wilcoxon test for non-parametric samples. Extracted texture features were then compared among FBP and all different ASIR-V levels, both with and without altering the spatial scale filter (SSF).

Statistical analysis was carried out using MedCalc (MedCalc Software, version15, Ostend, Belgium), and a *p* value < 0.05 was considered significant.

## 3. Results

### 3.1. Study Population

From an initial population of 75 patients enrolled, a total of 70 patients (31 males with a mean age of 65 years ± 14.48, and 39 females with a mean age of 67 years ± 15.4; age range, 39–92 years) were included, as depicted in Figure 1.

### 3.2. Data Reconstruction and CT Texture Analysis (CTTA) Analysis

A total of 770 datasets were obtained: in particular, a dataset of 70 reconstructions from FBP and 70 for each ASIR-V level (10% to 100%, with increments of 10%).

A first analysis was conducted comparing FBP reconstruction with each ASIR-V level reconstruction. Results showed significant differences for each structure examined (liver, kidney, spleen and paravertebral muscle) between FBP and all ASIR-V levels for mean intensity, entropy, SD and MPP (all *p* < 0.0001). In particular, the widest difference observed was for MPP extracted from muscle with a mean value of 33.52 ± 16.26 at FBP reconstruction and a value of 31.60 ± 17.85 extracted at ASIR-V 60% reconstruction. On the other hand, skewness and kurtosis showed no significant differences between FBP and all ASIR-V levels (all *p* > 0.05). Full results are summarized in Table 1 and Supplementary Table S1.

**Figure 4:**
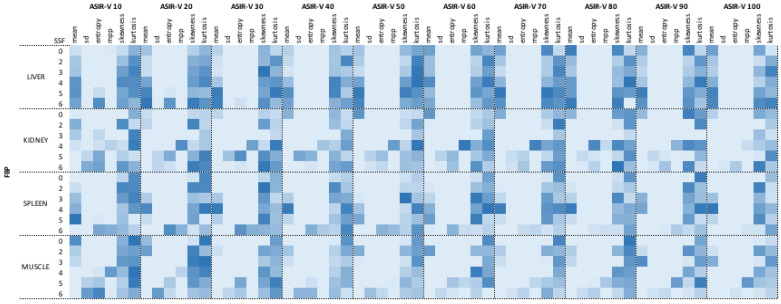
Heat map of filtered analysis. Dark blue boxes show the highest *p* values; light blue boxes the lowest.

Then, a second analysis was performed comparing FBP with all ASIR-V reconstructions for each SSF separately (SSF0, SSF2, SSF3, SSF4, SSF5, SSF6). The analysis confirmed the previous trend: significant differences between FBP and all ASIR-V reconstruction levels for mean intensity, SD, and MPP (all *p* < 0.0315), with the widest difference, observed for SD with SSF2 extracted from liver with a mean value of 36.10±8.26 at FBP reconstruction and mean value of 17.81 ± 4.75 extracted from ASIR-V 100% reconstruction. Entropy showed the same trend of significant differences, except for five comparisons that were non-significant at SSF5 and SSF6 extracted from low ASIR-V levels (10%, 20% and 30%). Regarding skewness and kurtosis, no significant differences were observed for all comparisons between FBP and all different ASIR-V datasets for each SSF (all *p* > 0.05). Full results for filtered (SSF0, SSF2, SSF3, SSF4, SSF5, SSF6) analysis of liver are summarized in Table 2. The heat map summarizes results for all filters for all organs segmented (Figure 4). Supplementary Tables S2–S5 summarize raw data of radiomic analysis. Supplementary Figure S1 shows a sample representative raw data distribution of SD, entropy, skewness and kurtosis of liver on SSF2.

## 4. Discussion

Our retrospective study demonstrated how some CTTA features of the liver, spleen and kidney parenchyma and of the paravertebral muscles were affected by different ASIR-V levels compared to FBP. In particular, mean intensity, SD, entropy and MPP were significantly affected by incremental ASIR-V levels, while no influence was reported for skewness and kurtosis. 

Considering the increasing number of CT examinations in several clinical scenarios [25], different vendors are paying more and more attention to the development of dose-reduction algorithms, trying to maintain at the same time an optimal image quality [26]. For this reason, the development and use of iterative reconstruction algorithms are significantly increasing [27,28,29]. Some studies pointed out how these different algorithms can influence the different radiomic features of CT images. An example is proposed by Meyer et al., who investigated how the confounding effect of differences in patient populations, acquisition parameters and reconstruction techniques among institutions can affect radiomic features and their reproducibility. In particular, they enrolled 78 patients known to have or suspected of having liver metastases from colon cancer (151 liver lesions were found), analyzing the number of reproducible radiomic features by modifying each technical parameter separately, keeping constant all the others. The percentage of radiomic features considered reproducible for any variation of different CT technical parameters (i.e., dose level, reconstructed section thickness, reconstruction kernel and algorithm) was 11.3%. The authors used for their study a different iterative reconstruction technique (SAFIRE), but their results have similarities with ours in terms of influence of different technical reconstruction parameters on radiomic features. In particular, they found that radiomic features in the intensity category (i.e., entropy, kurtosis, mean absolute deviation) are one of the most susceptible to changes in CT parameters, and these results are in line with our study (except for kurtosis), but discrepancies might be related to different IR applied [30]. 

Sung et al. [8] investigated the influence of three different reconstruction methods (FBP, hybrid IR (iDOSE) and model-based IR (IMR)) on CT first-order texture features of liver parenchyma in both normal and in chronic liver disease on scans acquired with contrast medium. CTTA was performed with the same software applied for our study. Their results showed that IR techniques affect various CT texture features (SD, entropy, skewness and kurtosis) of the healthy liver parenchyma in the same individuals across different filters. Discrepancies between the study of Sung and colleagues and our study underline how radiomics is still a technique that requires in-depth standardization processes. Similar results were found by Prezzi et al. [12], showing that incremental levels of ASIR significantly affect CT radiomics quantification in primary colorectal cancer compared to FBP. Their analysis was conducted using the CT acquisition corresponding to peak tumor enhancement, in order to maximize the tumor contrast-to-noise ratio on images reconstructed with 20% ASIR level increments, from 0 to 100%. Single-slice and multislice analyses were performed on first-order, second-order and high-order features. On single-slice analysis all first-order, second-order and fractal features varied significantly and according to a linear relationship with increasing ASIR values, with the exception of Grey-level co-occurrence matrix (GLCM) sum entropy. As in our results, although the study was carried out on tissue lesions, with a different reconstruction technique (ASIR vs ASIR-V) and on images obtained with contrast medium, it is possible to assume that the technique underlying the ASIR mechanism may have an influence on the first-order texture features regardless of the use of the contrast medium and whether the analysis is performed on a lesion or on healthy parenchyma. Moreover, Midya et al. in their study tried to assess the reproducibility of 248 radiomic features derived from computed tomography (CT) images performed on a uniform water phantom (UWP) and a human scan by varying some tools; they directed their attention to the adaptive statistical iterative reconstruction (ASIR) at different levels (0% to 100% with increments of 10%), considering the increasing attention paid to the dose-reduction issue. Their final data suggest that image acquisition parameters relating to image noise (i.e., tube current, noise index and reconstruction (ASIR)) strongly influence radiomic feature reproducibility precisely because features are based on the spatial distribution of pixel intensities. In particular, their study found that the number of reproducible features linearly decreased with increasing ASIR when compared with FBP (ASIR 0%) [31]. Although their iterative reconstruction technique was ASIR while ours was ASIR-V, and despite they performed their study on a phantom, our results were very similar. In particular, they extracted the same radiomic features that we analyzed in our study (including mean, standard deviation, skewness, kurtosis and entropy), and they established that increasing levels of ASIR affect radiomics features. These results were similar to our study, except for skewness and kurtosis, which in our study showed not to be significantly influenced by different ASIR-V levels compared with FBP. This is meaningful because we obtained similar results both in phantom and in human patients.

Despite the interesting results, our study has some limitations. First, it is a monocentric study with a small cohort (70 patients). Second, only the first-order texture features were extracted and analyzed. Third, our study was performed on images obtained from a single CT machine vendor and only on unenhanced images without considering the possible influence of the contrast medium. Finally, analysis was performed only on healthy structures without considering lesions or diffuse organ diseases.

## 5. Conclusions

In conclusion, the application of incremental ASIR-V levels versus traditional FBP affects CTTA across various filters. Skewness and kurtosis are not affected by iterative reconstructions and may be reliable quantitative parameters for radiomic analysis. Hence, for the validation of potential CT imaging biomarkers, image acquisition and reconstruction parameters must be harmonized to have reliable results.

## Figures and Tables

**Table 1:** Results of CT texture analysis (CTTA) of the liver (A), kidney (B), spleen (C) and muscle (D) on unfiltered images; each feature (standard deviation (SD), mean, entropy, mean of positive pixels (MPP), skewness and kurtosis) is compared between filtered back projection (FBP) and all Adaptive Statistical Iterative Reconstruction-V (ASIR-V) reconstructions. Significant *p* in bold.

*p* Value											
	Texture Features	FBP vs. ASIR 10	FBP vs. ASIR 20	FBP vs. ASIR 30	FBP vs. ASIR 40	FBP vs. ASIR 50	FBP vs. ASIR 60	FBP vs. ASIR 70	FBP vs. ASIR 80	FBP vs. ASIR 90	FBP vs. ASIR 100
**LIVER**	Mean	0.3086	0.0915	**0.0202**	**0.017**	0.1784	0.7772	0.1624	0.6789	0.1359	0.3816
	SD	**<0.0001**	**<0.0001**	**<0.0001**	**<0.0001**	**<0.0001**	**<0.0001**	**<0.0001**	**<0.0001**	**<0.0001**	**<0.0001**
	Entropy	**<0.0001**	**<0.0001**	**<0.0001**	**<0.0001**	**<0.0001**	**<0.0001**	**<0.0001**	**<0.0001**	**<0.0001**	**<0.0001**
	MPP	**<0.0001**	**<0.0001**	**<0.0001**	**<0.0001**	**<0.0001**	**<0.0001**	**<0.0001**	**<0.0001**	**<0.0001**	**<0.0001**
	Skewness	0.7458	0.9818	0.2423	0.4267	0.3224	0.5603	0.7819	0.7662	0.8089	0.8997
	Kurtosis	0.9544	0.3022	0.7583	0.4602	0.8883	0.8905	0.3393	0.6398	0.5437	0.2402
**KIDNEY**	Mean	**<0.0001**	**<0.0001**	**<0.0001**	**<0.0001**	**<0.0001**	**<0.0001**	**<0.0001**	**<0.0001**	**<0.0001**	**<0.0001**
	SD	**<0.0001**	**<0.0001**	**<0.0001**	**<0.0001**	**<0.0001**	**<0.0001**	**<0.0001**	**<0.0001**	**<0.0001**	**<0.0001**
	Entropy	**<0.0001**	**<0.0001**	**<0.0001**	**<0.0001**	**<0.0001**	**<0.0001**	**<0.0001**	**<0.0001**	**<0.0001**	**<0.0001**
	MPP	**<0.0001**	**<0.0001**	**<0.0001**	**<0.0001**	**<0.0001**	**<0.0001**	**<0.0001**	**<0.0001**	**<0.0001**	**<0.0001**
	Skewness	0.0004	0.0007	0.0014	0.0084	0.0076	0.0191	0.0189	0.0255	0.0455	0.0039
	Kurtosis	0.1911	0.4786	0.8986	0.5717	0.8302	0.782	0.5265	0.6984	0.5194	0.8733
**SPLEEN**	Mean	0.0173	0.5802	0.3894	0.4175	0.0143	0.0886	0.4117	0.7228	0.4932	0.6254
	SD	**<0.0001**	**<0.0001**	**<0.0001**	**<0.0001**	**<0.0001**	**<0.0001**	**<0.0001**	**<0.0001**	**<0.0001**	**<0.0001**
	Entropy	**<0.0001**	**<0.0001**	**<0.0001**	**<0.0001**	**<0.0001**	**<0.0001**	**<0.0001**	**<0.0001**	**<0.0001**	**<0.0001**
	MPP	**<0.0001**	**<0.0001**	**<0.0001**	**<0.0001**	**<0.0001**	**<0.0001**	**<0.0001**	**<0.0001**	**<0.0001**	**<0.0001**
	Skewness	0.2994	0.5019	0.8763	0.3017	0.1619	0.4204	0.878	0.3262	0.9454	0.7544
	Kurtosis	0.0274	0.0806	0.0666	0.3008	0.3777	0.2696	0.2733	0.3978	0.2961	0.8402
**MUSCLE**	Mean	**<0.0001**	**<0.0001**	**<0.0001**	**<0.0001**	**<0.0001**	**<0.0001**	**<0.0001**	**<0.0001**	**<0.0001**	**<0.0001**
	SD	**<0.0001**	**<0.0001**	**<0.0001**	**<0.0001**	**<0.0001**	**<0.0001**	**<0.0001**	**<0.0001**	**<0.0001**	**<0.0001**
	Entropy	**<0.0001**	**<0.0001**	**<0.0001**	**<0.0001**	**<0.0001**	**<0.0001**	**<0.0001**	**<0.0001**	**<0.0001**	**<0.0001**
	MPP	**<0.0001**	**<0.0001**	**<0.0001**	**<0.0001**	**<0.0001**	**<0.0001**	**<0.0001**	**<0.0001**	**<0.0001**	**<0.0001**
	Skewness	0.844	0.7554	0.5952	0.8766	0.8646	0.265	0.4455	0.6281	0.4013	0.3018
	Kurtosis	0.8118	0.9162	0.2451	0.5748	0.4268	0.5421	0.605	0.7219	0.9001	0.8274

**Table 2:** CTTA (CT texture analysis) of the liver on SSF0, SSF2, SSF3, SSF4, SSF5 and SSF6 filtered images; each feature (standard deviation (SD), mean, entropy, mean of positive pixels (MPP), skewness and kurtosis) is compared between filtered back projection (FBP) and all Adaptive Statistical Iterative Reconstruction-V (ASIR-V) reconstructions. Significant *p* in bold.

*p* Value											
SSF	Texture Features	FBP vs. ASIR 10	FBP vs. ASIR 20	FBP vs. ASIR 30	FBP vs. ASIR 40	FBP vs. ASIR 50	FBP vs. ASIR 60	FBP vs. ASIR 70	FBP vs. ASIR 80	FBP vs. ASIR 90	FBP vs. ASIR 100
**SSF0**	**Mean**	0.1073	0.3446	0.2228	0.2379	0.3423	0.6349	0.6235	0.9215	0.5029	0.6278
	**SD**	**<0.0001**	**<0.0001**	**<0.0001**	**<0.0001**	**<0.0001**	**<0.0001**	**<0.0001**	**<0.0001**	**<0.0001**	**<0.0001**
	**Entropy**	**<0.0001**	**<0.0001**	**<0.0001**	**<0.0001**	**<0.0001**	**<0.0001**	**<0.0001**	**<0.0001**	**<0.0001**	**<0.0001**
	**MPP**	**0.0315**	**0.0069**	**0.0009**	**0.0012**	**0.0043**	**0.0075**	**0.0098**	**0.0270**	**0.0077**	**0.0230**
	**Skewness**	0.1495	0.2402	0.4741	0.3372	0.4219	0.6082	0.8381	0.8545	0.8677	0.6025
	**Kurtosis**	0.4749	0.4437	0.1814	0.2282	0.5967	0.4581	0.1644	0.1711	0.1790	0.0724
**SSF2**	**Mean**	0.3152	0.1430	0.1007	0.159	0.2756	0.3685	0.3174	0.4117	0.2582	0.3152
	**SD**	**<0.0001**	**<0.0001**	**<0.0001**	**<0.0001**	**<0.0001**	**<0.0001**	**<0.0001**	**<0.0001**	**<0.0001**	**<0.0001**
	**Entropy**	**<0.0001**	**<0.0001**	**<0.0001**	**<0.0001**	**<0.0001**	**<0.0001**	**<0.0001**	**<0.0001**	**<0.0001**	**<0.0001**
	**MPP**	**<0.0001**	**<0.0001**	**<0.0001**	**<0.0001**	**<0.0001**	**<0.0001**	**<0.0001**	**<0.0001**	**<0.0001**	**<0.0001**
	**Skewness**	**0.377**	0.5052	0.4475	0.322	0.1715	0.2035	0.2735	0.2538	0.2116	0.1208
	**Kurtosis**	0.7801	0.5771	0.7402	0.8523	0.8991	0.8574	0.733	0.7566	0.7142	0.5273
**SSF3**	**Mean**	0.3433	0.126	0.0668	0.0662	0.1475	0.3848	0.1408	0.3116	0.1803	0.2582
	**SD**	**<0.0001**	**<0.0001**	**<0.0001**	**<0.0001**	**<0.0001**	**<0.0001**	**<0.0001**	**<0.0001**	**<0.0001**	**<0.0001**
	**Entropy**	**<0.0001**	**<0.0001**	**<0.0001**	**<0.0001**	**<0.0001**	**<0.0001**	**<0.0001**	**<0.0001**	**<0.0001**	**<0.0001**
	**MPP**	**<0.0001**	**<0.0001**	**<0.0001**	**<0.0001**	**<0.0001**	**<0.0001**	**<0.0001**	**<0.0001**	**<0.0001**	**<0.0001**
	**Skewness**	0.6375	0.6069	0.9964	0.3372	0.2858	0.2441	0.2715	0.4207	0.2717	0.4429
	**Kurtosis**	0.8737	0.7554	0.4551	0.2856	0.9875	0.6472	0.8629	0.5503	0.4836	0.888
**SSF4**	**Mean**	0.7373	0.6044	0.3162	0.3936	0.6122	0.8156	0.4591	0.687	0.3251	0.5119
	**SD**	**<0.0001**	**<0.0001**	**<0.0001**	**<0.0001**	**<0.0001**	**<0.0001**	**<0.0001**	**<0.0001**	**<0.0001**	**<0.0001**
	**Entropy**	**<0.0001**	**<0.0001**	**<0.0001**	**<0.0001**	**<0.0001**	**<0.0001**	**<0.0001**	**<0.0001**	**<0.0001**	**<0.0001**
	**MPP**	**<0.0001**	**<0.0001**	**<0.0001**	**<0.0001**	**<0.0001**	**<0.0001**	**<0.0001**	**<0.0001**	**<0.0001**	**<0.0001**
	**Skewness**	0.8109	0.6822	0.6839	0.8761	0.6464	0.6357	0.4522	0.3845	0.4406	0.223
	**Kurtosis**	0.81	0.8385	0.7265	0.531	0.9949	0.9386	0.6713	0.992	0.9502	0.5297
**SSF5**	**Mean**	0.7471	0.8662	0.9327	0.7856	0.9583	0.4163	0.9723	0.693	0.9299	0.8848
	**SD**	**<0.0001**	**<0.0001**	**<0.0001**	**<0.0001**	**<0.0001**	**<0.0001**	**<0.0001**	**<0.0001**	**<0.0001**	**<0.0001**
	**Entropy**	0.1436	0.0517	**<0.0001**	**<0.0001**	**<0.0001**	**<0.0001**	**<0.0001**	**<0.0001**	**<0.0001**	**<0.0001**
	**MPP**	**0.0033**	**<0.0001**	**<0.0001**	**<0.0001**	**<0.0001**	**<0.0001**	**<0.0001**	**<0.0001**	**<0.0001**	**<0.0001**
	**Skewness**	0.5869	0.4436	0.591	0.8702	0.9113	0.7306	0.9732	0.7433	0.7151	0.6131
	**Kurtosis**	0.7911	0.4026	0.9569	0.2716	0.7697	0.5892	0.8379	0.6917	0.5408	0.4489
**SSF6**	**Mean**	0.6203	0.9877	0.9117	0.6203	0.8652	0.5796	0.765	0.9495	0.7564	0.8552
	**SD**	**<0.0001**	**<0.0001**	**<0.0001**	**<0.0001**	**<0.0001**	**<0.0001**	**<0.0001**	**<0.0001**	**<0.0001**	**<0.0001**
	**Entropy**	0.7935	0.7242	0.0889	**<0.0001**	**<0.0001**	**<0.0001**	**<0.0001**	**<0.0001**	**<0.0001**	**<0.0001**
	**MPP**	**0.0097**	**0.0045**	**0.0001**	**<0.0001**	**<0.0001**	**<0.0001**	**<0.0001**	**<0.0001**	**<0.0001**	**<0.0001**
	**Skewness**	0.6368	0.9408	0.7817	0.4941	0,5471	0.3881	0.8335	0.9179	0.7651	0.8109
	**Kurtosis**	0.4555	0.7421	0.4264	0.6368	0.6527	0.7013	0.7392	0.8953	0.3394	0.8918

## Data Availability

Data supporting results can be provided by the corresponding author A.L.

## Supplementary Materials

The following are available online at https://www.mdpi.com/article/10.3390/diagnostics11061000/s1, Figure S1: sample representative raw data distribution of standard deviation (SD), entropy, skewness and kurtosis of liver on Spatial Scaling Factor 2. Table S1: results of CT texture analysis of the liver, kidney, spleen and muscle on unfiltered images expressed as mean and standard deviation. Table S2: raw data of CT texture analysis of the liver. Table S3: raw data of CT texture analysis of the kidney. Table S4: raw data of CT texture analysis of the muscle. Table S5: raw data of CT texture analysis of the spleen.
