# Supplementary material for: Influence of Adaptive Statistical Iterative Reconstructions on CT Radiomic Features in Oncologic Patients
Source: Diagnostics (Basel). 2021 May 31;11(6):1000. doi: 10.3390/diagnostics11061000 (PMC8229560; doi:10.3390/diagnostics11061000)
Supplement: Supplementary file 1 [file diagnostics-11-01000-s001.zip › Supplementary Table S1.pdf]

Table S1. Mean and Standard Deviation -SD- of CTTA (CT Texture Analysis) of the Liver, Kidney, Spleen and Muscle for each feature (Mean. Entropy, Mean of Positive Pixels -MPP-, Skewness and Kurtosis).

	Mean and Standard Deviation											
	**SSF**	**FBP**	**ASIR 10**	**ASIR 20**	**ASIR 30**	**ASIR 40**	**ASIR 50**	**ASIR 60**	**ASIR 70**	**ASIR 80**	**ASIR 90**	**ASIR 100**
**LIVER**	**Mean**	10.24 ± 21.68	10.23 ± 21.66	10.18 ± 21.68	10.17 ± 21.67	10.15 ± 21.67	10.16 ± 21.64	10.21 ± 21.62	10.17 ± 21.63	10.19 ± 21.59	10.13 ± 21.60	10.19 ± 21.59
	**SD**	18.47 ± 10.91	17.39 ± 10.04	16.58 ± 9.43	15.75 ± 8.86	14.94 ± 8.37	14.19 ± 7.91	13.60 ± 7.58	12.99 ± 7.24	12.37 ± 6.92	11.78 ± 6.67	11.38 ± 6.58
	**Entropy**	3.78 ± 0.44	3.75 ± 0.42	3.73 ± 0.42	3.69 ± 0.41	3.65 ± 0.41	3.62 ± 0.40	3.59 ± 0.39	3.56 ± 0.39	3.52 ± 0.39	3.48 ± 0.40	3.44 ± 0.41
	**MPP**	22.81 ± 17.96	22.18 ± 17.86	21.69 ± 17.83	21.18 ± 17.79	20.71 ± 17.85	20.27 ± 17.86	19.95 ± 17.89	19.54 ± 17.96	19.16 ± 18.00	18.78 ± 18.08	18.57 ± 18.14
	**Skewness**	0.01 ± 0.45	0.01 ± 0.45	0.01 ± 0.45	0.01 ± 0.46	0.01 ± 0.47	0.01 ± 0.47	0.01 ± 0.47	0.01 ± 0.48	0.01 ± 0.49	0.01 ± 0.49	0.01 ± 0.49
	**Kurtosis**	-0.35 ± 0.61	-0.35 ± 0.63	-0.34 ± 0.66	-0.34 ± 0.63	-0.33 ± 0.65	-0.34 ± 0.65	-0.34 ± 0.64	-0.34 ± 0.65	-0.33 ± 0.66	-0.34 ± 0.66	-0.35 ± 0.66
**KIDNEY**	**Mean**	14.82 ± 17.99	15.05 ± 18.05	15.35 ± 18.02	15.58 ± 18.01	15.77 ± 18.15	16.01 ± 17.89	16.05 ± 18.11	16.17 ± 18.17	16.28 ± 18.21	16.59 ± 18.08	16.52 ± 18.00
	**SD**	22.24 ± 13.44	21.13 ± 12.94	20.35 ± 12.60	19.61 ± 12.33	18.92 ± 12.17	18.14 ± 11.64	17.67 ± 11.94	17.12 ± 11.91	16.54 ± 11.90	16.14 ± 11.98	15.51 ± 11.52
	**Entropy**	3.78 ± 0.36	3.76 ± 0.35	3.73 ± 0.34	3.71 ± 0.34	3.69 ± 0.34	3.67 ± 0.33	3.65 ± 0.34	3.62 ± 0.35	3.59 ± 0.36	3.56 ± 0.38	3.53 ± 0.40
	**MPP**	26.74 ± 11.13	26.22 ± 11.12	25.93 ± 10.89	25.61 ± 10.85	25.37 ± 10.99	25.07 ± 11.08	24.82 ± 11.16	24.59 ± 11.30	24.33 ± 11.52	24.31 ± 11.62	23.92 ± 11.84
	**Skewness**	-0.03 ± 0.56	-0.02 ± 0.56	-0.02 ± 0.57	-0.02 ± 0.57	-0.02 ± 0.57	-0.01 ± 0.57	-0.02 ± 0.57	-0.02 ± 0.58	-0.02 ± 0.59	-0.02 ± 0.59	0.00 ± 0.60
	**Kurtosis**	-0.26 ± 0.96	-0.26 ± 0.99	-0.25 ± 1.00	-0.25 ± 1.02	-0.25 ± 1.04	-0.24 ± 1.05	-0.25 ± 1.05	-0.24 ± 1.06	-0.23 ± 1.09	-0.24 ± 1.08	-0.23 ± 1.09
**SPLEEN**	**Mean**	11.53 ± 21.86	11.29 ± 21.91	11.31 ± 21.88	11.32 ± 21.86	11.33 ± 21.82	11.30 ± 21.82	11.30 ± 21.82	11.34 ± 21.79	11.40 ± 21.77	11.44 ± 21.74	11.34 ± 21.85
	**SD**	18.39 ± 11.08	17.28 ± 10.13	16.49 ± 9.56	15.75 ± 9.06	15.00 ± 8.65	14.23 ± 8.14	13.74 ± 7.91	13.08 ± 7.61	12.48 ± 7.37	11.94 ± 7.18	11.54 ± 7.16
	**Entropy**	3.69 ± 0.44	3.67 ± 0.42	3.64 ± 0.42	3.62 ± 0.41	3.58 ± 0.41	3.55 ± 0.40	3.53 ± 0.40	3.49 ± 0.40	3.45 ± 0.41	3.42 ± 0.41	3.38 ± 0.42
	**MPP**	23.15 ± 16.85	22.38 ± 16.73	21.92 ± 16.70	21.50 ± 16.67	21.08 ± 16.70	20.62 ± 16.73	20.31 ± 16.79	19.97 ± 16.85	19.62 ± 16.95	19.32 ± 17.01	19.02 ± 17.12
	**Skewness**	-0.05 ± 0.53	-0.05 ± 0.54	-0.05 ± 0.55	-0.05 ± 0.55	-0.05 ± 0.55	-0.04 ± 0.55	-0.04 ± 0.56	-0.05 ± 0.56	-0.04 ± 0.57	-0.05 ± 0.57	-0.06 ± 0.58
	**Kurtosis**	-0.21 ± 0.83	-0.21 ± 0.83	-0.21 ± 0.85	-0.21 ± 0.84	-0.21 ± 0.84	-0.20 ± 0.85	-0.20 ± 0.86	-0.19 ± 0.88	-0.18 ± 0.89	-0.18 ± 0.89	-0.20 ± 0.89
**MUSCLE**	**Mean**	21.50 ± 23.79	21.81 ± 23.95	21.87 ± 23.95	22.04 ± 23.99	22.14 ± 24.13	22.29 ± 24.15	22.33 ± 24.26	22.41 ± 24.13	22.41 ± 24.32	22.29 ± 24.25	22.30 ± 24.27
	**SD**	24.21 ± 13.38	23.32 ± 12.88	22.74 ± 12.80	21.82 ± 12.43	21.11 ± 12.26	20.38 ± 12.09	19.75 ± 11.98	19.09 ± 11.65	18.57 ± 11.87	17.97 ± 11.85	17.49 ± 11.86
	**Entropy**	3.90 ± 0.38	3.88 ± 0.38	3.86 ± 0.38	3.84 ± 0.38	3.81 ± 0.37	3.79 ± 0.38	3.77 ± 0.38	3.74 ± 0.39	3.71 ± 0.40	3.68 ± 0.42	3.65 ± 0.43
	**MPP**	33.52 ± 16.26	33.22 ± 16.36	32.99 ± 16.54	32.54 ± 16.73	32.24 ± 17.01	31.92 ± 17.20	31.60 ± 17.49	31.28 ± 17.69	30.98 ± 17.89	30.55 ± 18.01	30.24 ± 18.11
	**Skewness**	-0.15 ± 0.51	-0.14 ± 0.52	-0.15 ± 0.52	-0.15 ± 0.52	-0.14 ± 0.53	-0.14 ± 0.54	-0.14 ± 0.54	-0.14 ± 0.54	-0.14 ± 0.55	-0.13 ± 0.56	-0.14 ± 0.56
	**Kurtosis**	-0.25 ± 0.82	-0.25 ± 0.83	-0.24 ± 0.85	-0.25 ± 0.87	-0.23 ± 0.90	-0.23 ± 0.90	-0.23 ± 0.90	-0.23 ± 0.92	-0.23 ± 0.92	-0.22 ± 0.94	-0.23 ± 0.94
