# Supplementary figures and images for: Influence of Adaptive Statistical Iterative Reconstructions on CT Radiomic Features in Oncologic Patients
Source: Diagnostics (Basel). 2021 May 31;11(6):1000. doi: 10.3390/diagnostics11061000 (PMC8229560; doi:10.3390/diagnostics11061000)

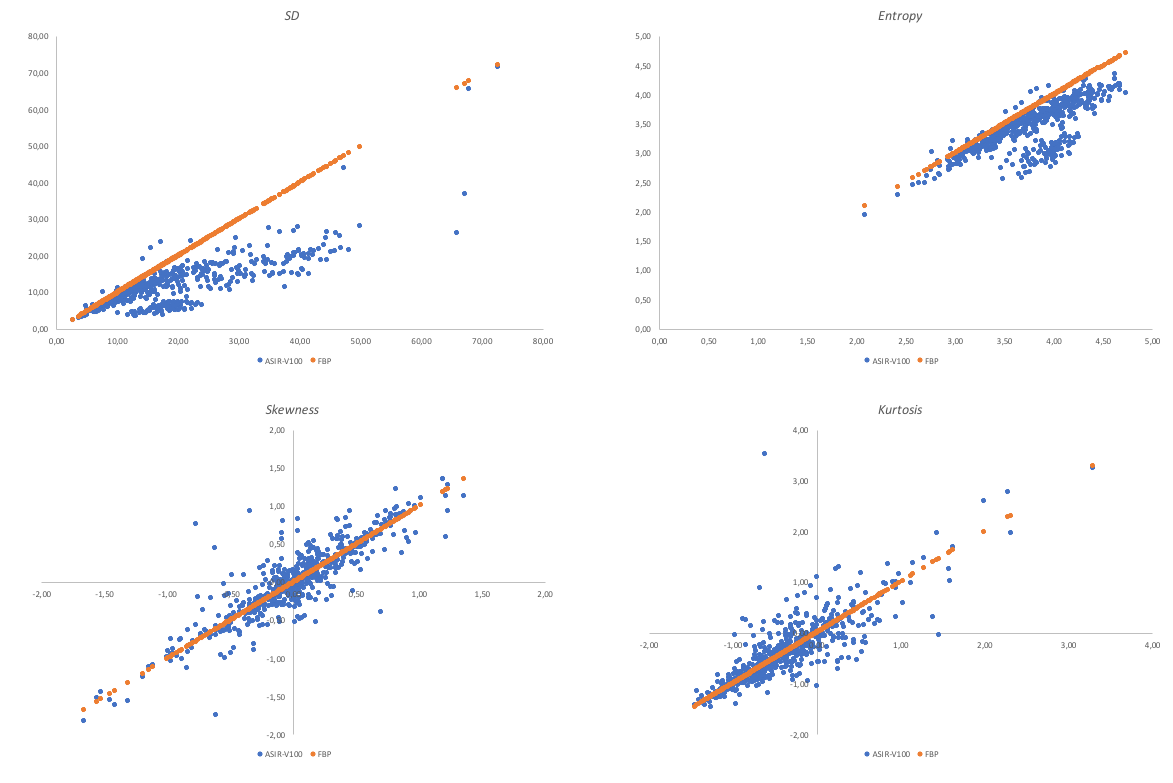

Supplement: Supplementary file 1 [file diagnostics-11-01000-s001.zip › Supplementary Figure S1.tiff]
